# Effects of positive and negative social feedback on motivation, evaluative learning, and socio-emotional processing

**DOI:** 10.1038/s41539-023-00178-7

**Published:** 2023-08-16

**Authors:** Alexandra Sobczak, Nico Bunzeck

**Affiliations:** 1 Department of Psychology, University of Lübeck, Ratzeburger Allee 160, 23562 Lübeck, Germany; 2 Center of Brain, Behavior and Metabolism, University of Lübeck, Ratzeburger Allee 160, 23562 Lübeck, Germany

**Keywords:** Human behaviour, Psychology

## Abstract

Social rewards and punishments are strong motivators. Since experimental work has focused on young adults using simplistic feedback, the effects of more naturalistic stimuli on motivation, evaluative learning, and socio-emotional processing with advanced age remain unclear. Therefore, we compared the effects of static (photos) vs dynamic (videos) social feedback in a social incentive delay (SID) task in young (18–35 years) and older adults (50–84 years) with neutral, positive, and negative feedback, on response times (RTs), and assessed the emotional valence of feedback cues and feedback videos. We found that anticipating positive and negative social feedback accelerated RTs regardless of age and without additional effects of video feedback. Furthermore, the results suggest a valence transfer from positive feedback videos to predictive cues in both groups (i.e., evaluative learning). Finally, older adults reported less pronounced negative affect for negative feedback videos, indicating age differences in socio-emotional processing. As such, our findings foster our understanding of the underlying cognitive and emotional aspects involved in the processing of social rewards and punishments.

## Introduction

Social encounters in the real world are multimodal but our scientific understanding of social information processing is often based on abstract paradigms and simplistic stimuli in highly controlled experiments. While this approach provides high internal validity, it comes at the cost of external validity and generalizability with respect to real world social interactions. In fact, real-life social information is much richer due to visual, semantic, and prosodic aspects, as well as dynamic properties, i.e., it involves serial and simultaneous information^1^. Although recent advances in social psychology and cognitive neuroscience try to bridge this gap by using more sophisticated experimental designs^2,3^, the effects of more naturalistic social feedback on motivation, learning, and socio-emotional processing and possible age-related changes are still poorly understood. For example, age-related changes in emotional memory^4^ and valence judgement of emotional faces^5^ have been shown with static stimuli, but the effect of more realistic social information remains unclear. To address these open questions, we compared the effects of static (photos) vs dynamic (pre-recorded videos) social feedback on response times (RTs) as well as the subjective emotional valence of feedback predicting cues and feedback stimuli in young (18–35 years) and older (50–84 years) adults using the social incentive delay (SID) task. In this variation of the widely used monetary incentive delay (MID) task^6^, faster RTs are used as an indicator of increased motivation in a reinforcement learning context.

The prospect of reward and punishment leads to changes in motivation and behavior. This effect has been reported in studies using different types of feedback, including written texts^7^, social photos^8,9^, social videos of faces and gestures^10^, social videos of body movements^7^, as well as monetary incentives^11–13^. Generally, RTs are faster when anticipating high vs low monetary and social rewards^8,14^ as well as positive and negative vs neutral social feedback ^9,10^. While videos possess more engaging properties^15,16^, and natural human body movement (also called biological motion) is also valued more than rigid machine-like motion^17^, viewing body movement video feedback without faces does not lead to faster RT compared to text feedback in the SID task^7^. Thus, biological motion alone appears not to be a stronger motivator, but a feeling of social presence may be critical. For instance, video feedback in online teaching creates a greater social presence of the teacher than written text and it helps to perceive the teacher as a real person^18,19^. However, it is unknown whether videos including biological motion in mimics and gestures plus verbal feedback leads to faster RTs vs photos showing static mimics and gestures since the two variants of social feedback have not been compared directly.

Rewards and punishments are associated with pleasant and unpleasant affect, respectively. Stimuli that predict rewards and punishments have been argued to acquire predictive value (expectancy learning) and emotional valence (evaluative learning)^20^. Evaluative learning refers to the transfer of emotional valence from the valent to the initially neutral stimulus and occurs when a neutral and a valent stimulus are repeatedly paired^21^. Accordingly, the mental representation of the stimulus is changed, which can lead to biases in perception, thoughts, or actions^22^. Indeed, verbal ratings of human faces changed from neutral to negative after contingent pairing with unpleasant but tolerable electrical stimulation in an aversive conditioning paradigm^20^. While the SID and MID task have been used to study motivational changes and neural correlates associated with anticipating rewards and punishments, behavioral measures of the emotional valence of the predictive cues are scarce and evidence in favor of evaluative learning in the context of social reward and punishment predicting cues as well as possible age effects remains elusive.

Learning from reward and punishment seems to be impaired with age to some degree, and neuroimaging studies suggest a link to compromised updating of predictive value^23,24^. In fact, aging is associated with the neural degeneration of the dopaminergic mesolimbic system^25,26^, which provides the key brain structures implicated in learning^27^. In terms of motivation, a reduced anticipation of positive or negative events would also impair goal-directed behavior. For instance, older adults (66–86 years) showed worse performance than younger adults (19–33 years) in decision tasks that require probabilistic learning to optimize reward outcome^23,28^. Importantly, in less demanding settings, such as the SID task, the motivational effect of anticipating rewards and punishments, reflected in faster RTs, is preserved in older adults (51–78 years)^8,9^ in spite of typical age-related motor slowing^9,29^.

Being judged by others is associated with emotional responses and emotional processing of social stimuli changes with age. For instance, empirical studies reported the so-called positivity effect, which was expressed in lower scores of negative affect (59–69 < 29–57 < 19–28 years) and less negative ratings of negative and neutral faces (19–69 years)^5^, increased attention and better memory for positive faces (60–94 > 18–35 years)^30^, reduced amygdala activation to negative emotional facial expression (62–72 < 19–39 years)^31^, and reduced neural responses to regret in a gambling paradigm (mean age 65 years < 25 years)^32^. Stronger neural responses to cues predicting social than monetary reward in older (60–78 years) compared to young (20–28 years) adults^8^ suggest that age-related changes in socio-emotional processing play a role in the SID task. A theoretical framework to explain these effects is the socioemotional selectivity theory (SST), which suggests that old age is associated with a stronger realization that the remaining life time is limited, leading to motivational changes such as a stronger focus on emotional goals^33^. Moreover, substantial age effects in recognizing the emotional valence of facial expressions also depend on the dynamic properties of the employed stimuli, as shown in a large cross-sectional study. Here, older adults performed significantly worse in the recognition of emotions in static images while recognition of dynamic facial expressions was relatively stable (over a period of 30 years from 61–90)^34^. Together, aging is associated with changes in socio-emotional processing but the interaction with dynamic social feedback and a possible relationship in motivational settings remains unclear.

Addressing the effects of dynamic social feedback on basic processes of motivation, learning, and emotion may lead to insights that could have direct consequences in educational contexts. For instance, digital learning tools are increasingly used and implementing dynamic social feedback could potentially increase the motivation to perform well on a task, e.g., studying vocabularies with an app. Moreover, the expectation of a pleasurable learning experience via dynamic social feedback could increase learning effort such as frequency or duration of interaction with the learning material. As a result, learning success could be enhanced. Further, concerning adult education, it would be beneficial to understand possible age-related changes.

**Fig. 1:**
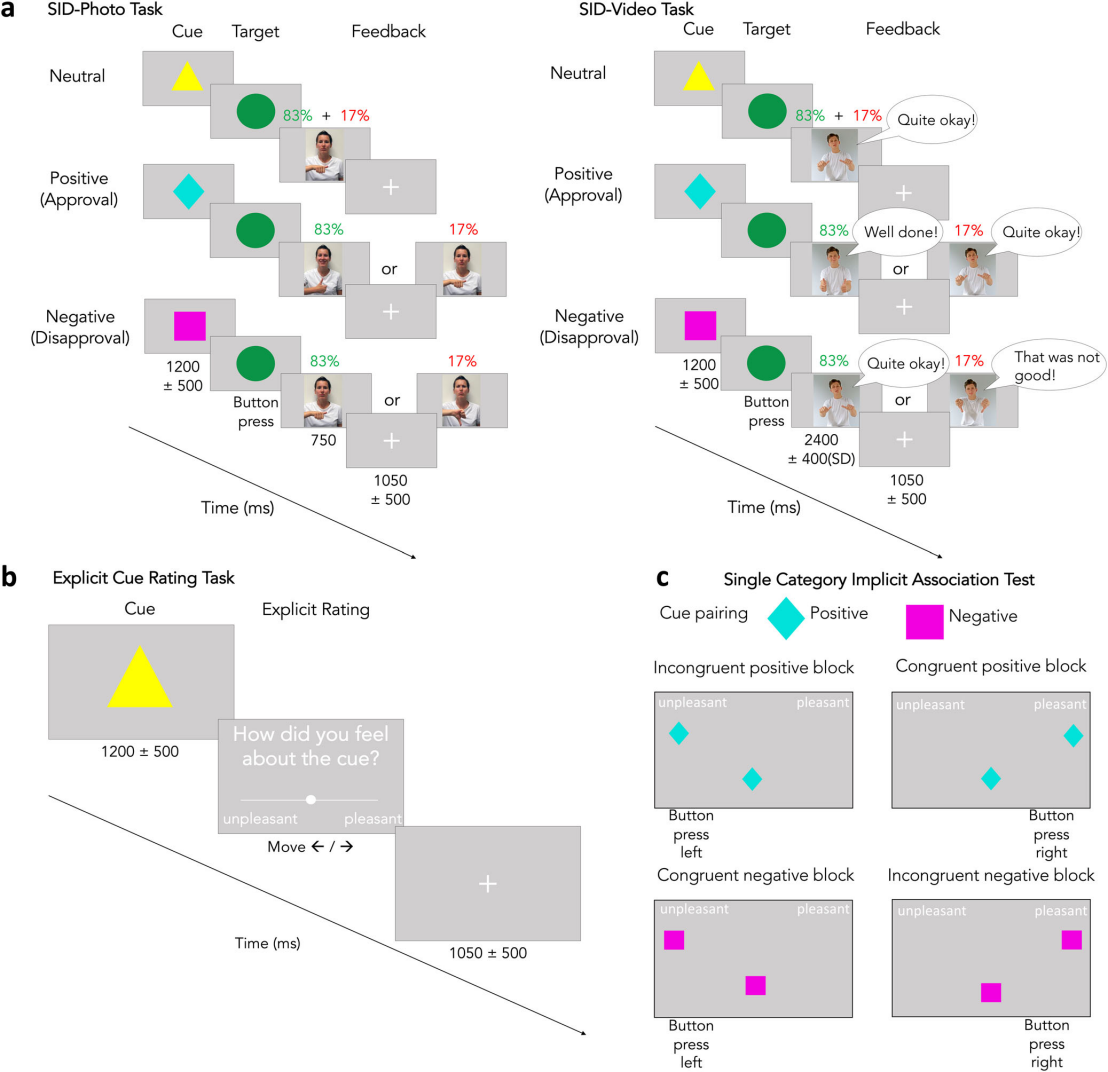
Schematic illustrations of experimental tasks. **a** Social incentive delay (SID) task. Feedback on task performance (i.e., RT) was given via photos or videos also including verbal feedback. Task difficulty was adapted from trial to trial achieving an average hit rate of 83% using a staircase method. In the positive and negative condition, the given feedback depended on hits (green) vs misses (red) while feedback in the neutral condition was always neutral. **b** Trial design in the explicit cue rating task. **c** Example trials for each block in the Single Category Implicit Association Test (SC-IAT). The bottom texts indicate the correct response to the target presented in the center of the screen (left indicates pressing the x-key and right indicates pressing the m-key).

To investigate the effects of dynamic social feedback on motivation, evaluative learning, and socio-emotional processing in aging, we performed two behavioral studies with young (*n* = 101; mean age 23 years; age range 18–35 years) and older adults (*n* = 107; mean age 64 years; age range 50–84 years) using the SID task with three conditions: neutral, positive, and negative social feedback (Fig. 1a). The first study used static (photos) and the second study dynamic (videos with audio) social feedback. Data in the first study was reused from a previously published study in which we aimed to assess motivational differences between Parkinson’s disease patients as well as healthy older and young adults^9^. Specifically, we reused the behavioral data from the young and older healthy controls from a simple RT task and the SID task. The SID comprises three phases: cue, response to the target, and feedback. The cues signaled the condition and the potential feedback to be received. Obtaining rewards and avoiding punishment depended on RT to the target. We assessed baseline RT for each participant in a separate simple RT task prior to conducting the SID. In study two, we also examined the emotional valence of the predictive cues by a direct and an indirect measure to assess evaluative learning. The direct measure was an explicit rating task (Fig. 1b) and implemented at the end of the SID task by adding extra trials. Here, the cue was followed by a screen instead of the target, asking the participants to rate their emotion on the previously presented cue. The indirect measure was an adapted version of the Implicit Association Test (IAT)^35^ for single categories (SC-IAT; Fig. 1c)^36^. In the last part of study two, we evaluated the emotional processing of the social video feedback stimuli by presenting them to the participants and asking them to rate the emotional valence (from negative to positive). Further, credibility (in the sense of authenticity and naturality) of the feedback stimuli was assessed in the last part. See the methods section for more details on the tasks.

Based on our previous findings^9^, we expected faster RTs for young and older adults following positive and negative cues (as compared to neutral cues), which was hypothesized to be more pronounced for videos vs photos as social feedback. We further expected emotional cue valence to correspond with the valence of the associated feedback (i.e., more positive evaluations for cues associated with positive feedback in contrast to cues associated with neutral and negative feedback). Finally, we predicted a positivity effect (i.e., more positive evaluations in older adults) and a positive correlation between cue valence and video feedback valence independent of age.

## Results

### Simple RT task

**Fig. 2:**
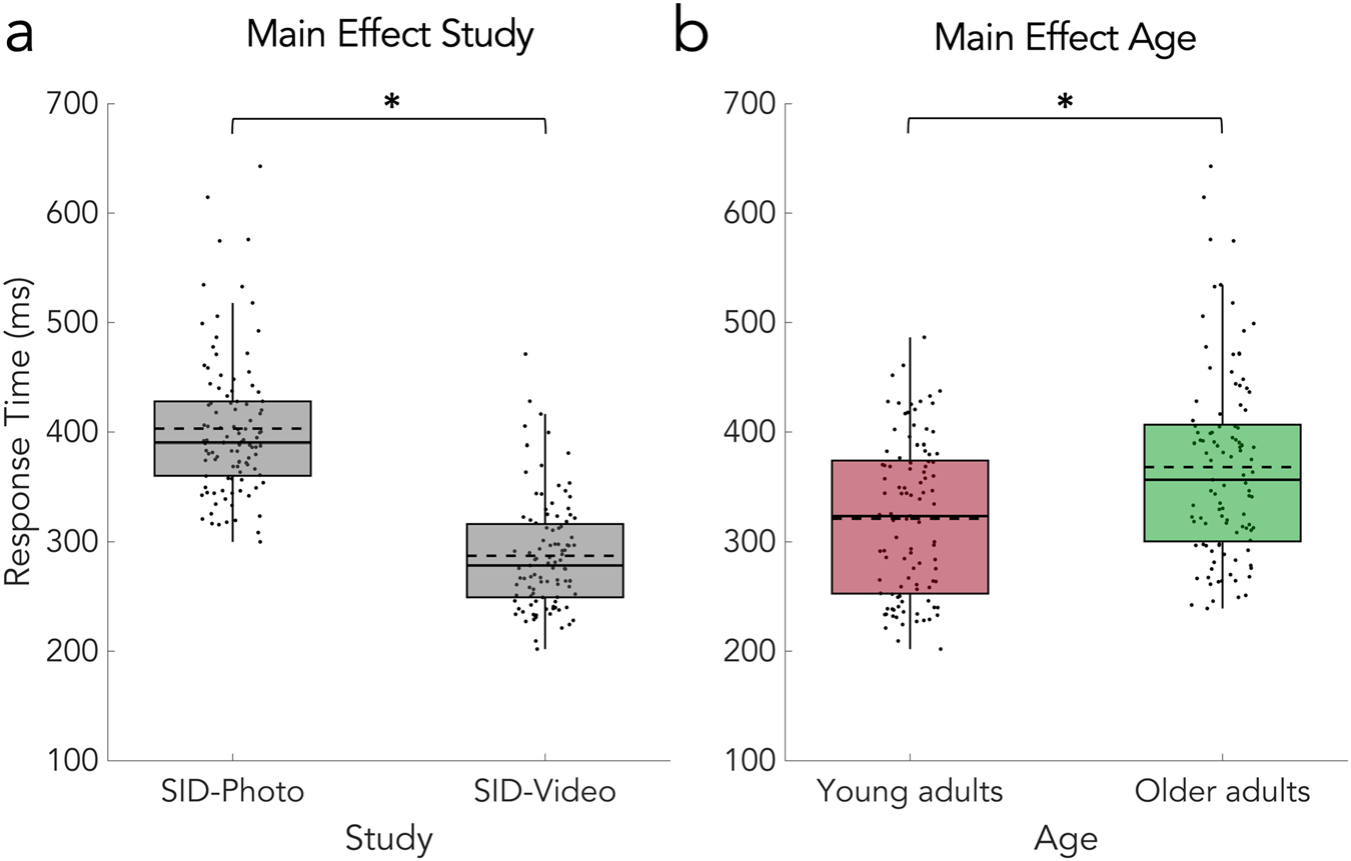
Results of the simple RT task in the SID-Photo and SID-Video study. **a** RT was significantly slower in the SID-Photo study than in the SID-Video study (main effect of study) and **b** young adults responded significantly faster than older adults (main effect of age). Statistical comparisons were made using a 2 × 2 design (study x age) permutation *F*-test. Data is presented in boxplots overlaid with data points. Boxes span from the lower to the upper quantile and whiskers of the boxplots depict data points that are the furthest from the center while still being inside the range of 1.5 times the interquartile range from the lower or upper quartile. Solid lines indicate the median, dashed lines indicate the mean. Asterisks mark significant differences (*p* < 0.001).

In the simple RT task, participants had to respond to a target as fast as possible. RTs were used to adjust the SID task to the individual response speed and analyzed to inspect possible baseline differences between the SID-Photo and SID-Video study. The average RT in the SID-Photo study was 379 ms (SD = 40 ms) for young adults and 427 ms (SD = 74 ms) for older adults. In the SID-Video study, the average RT was 259 ms (SD = 33 ms) for young adults and 312 ms (SD = 50 ms) for older adults. The 2 × 2 design (study x age) permutation *F*-test on RT in the simple RT task showed a significant main effect of study (*F*_(1,202)_ = 257.9, *p* = 0.0001, CI 95% = [−0.0001 0.0005], η^2^_p_ = 0.5608; Fig. 2a) and a significant main effect of age (*F*_(1,202)_ = 47.9, *p* = 0.0001, CI95% = [-0.0001 0.0005], η^2^_p_ = 0.1919; Fig. 2b), but no significant interaction (*F*_(1,202)_ = 0.1, *p* = 0.6663, CI 95% = [0.6532, 0.6793], η^2^_p_ = 0.0009).

### RT in SID-Photo and SID-Video

In the SID task, participants had to respond to a target as fast as possible with faster RTs being interpreted as increased motivation. The target was preceded by a cue signaling the condition (neutral, positive, negative) and potential feedback to be received (see Fig. 1a). In the positive condition, positive feedback was received for fast responses; in the negative condition, negative feedback was received for slow responses; and, in the neutral condition the feedback was always neutral. The SID-Photo task used images of volunteers showing neutral, positive, or negative mimics and gestures; and the SID-Video task used videos of volunteers showing neutral, positive, and negative mimics, gestures, and giving verbal feedback. RT was z-transformed to account for baseline differences in RT between studies.

**Fig. 3:**
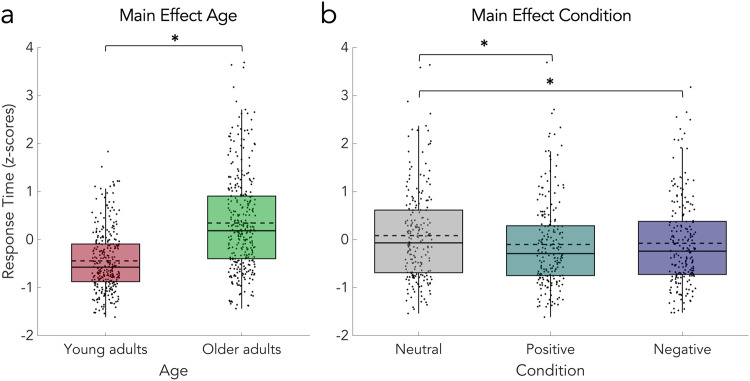
Results of the SID-Photo and SID-Video tasks. **a** Significant main effect of age. RTs are z-transformed. **b** Significant main effect of condition and significant differences between positive vs neutral and negative vs neutral but not positive vs negative condition. Statistical comparisons were made using a 2 × 2 × 3 mixed design (between factors study and age; within-factor condition) permutation *F*-test and two-sided permutation paired *t*-tests (with Bonferroni correction). Data are presented in boxplots overlaid with data points. Boxes span from the lower to the upper quantile and whiskers of the boxplots depict data points that are the furthest from the center while still being inside the range of 1.5 times the interquartile range from the lower or upper quartile. Solid lines indicate the median, dashed lines indicate the mean. Asterisks mark significant differences (*p* < 0.001).

In both tasks, z-transformed RT was faster for young (SID-Photo: Neutral: M = −0.36, SD = 0.72; Positive; M = −0.54, SD = 0.60; Negative: M = −0.50, SD = 0.64; SID-Video: Neutral: M = −0.27, SD = 0.65; Positive: M = −0.48, SD = 0.56; Negative: M = −0.48, SD = 0.59) than for older (SID-Photo: Neutral: M = 0.50, SD = 0.96; Positive; M = 0.36, SD = 1.01; Negative: M = 0.34, SD = 0.90; SID-Video: Neutral: M = 0.40, SD = 1.08; Positive: M = 0.19, SD = 0.96; Negative: M = 0.26, SD = 1.04) adults. The 2 × 2 × 3 mixed design permutation *F*-test showed significant main effects of age (*p* = 0.0001, Fig. 3a) and condition (*p* = 0.0001; Fig. 3b) but no significant main effect of study and no significant interactions. Post-hoc permutation paired *t*-tests revealed a significant difference between the positive vs and negative vs neutral (*p* = 0.0001) but not positive vs negative condition (Fig. 3b). See Table 1 for all statistical details. In line with these results, the 2 × 2 × 3 mixed Bayesian ANOVA provided extreme evidence for the main effects model with the factors age and condition (age + condition BF_10_ = 2.44 × 10^13^), which outperformed all other models. Moreover, the analysis provided strong evidence against the interaction of age and condition (age + condition BF_10_/age + condition + age * condition BF_10_ = 2.44 × 10^13^/1.01 × 10^12^ = 24.15). This means that the data is 24.15 times more likely under the two main effects model than under the model that includes their interaction. Table 2 provides an overview of all Bayes factors for the full model comparison.

**Table 1:** Results of the social incentive delay (SID) task.

2 × 2 × 3 *F*-test	df	*F*	*p*	CI 95%	η2p
Main effect study	1, 201	0.05	0.8134	[0.8026, 0.8242]	0.0002
Main effect age	1, 201	51.15	0.0001	[−0.0001, 0.0005]	0.2029
Main effect condition	2, 402	14.62	0.0001	[−0.0001, 0.0005]	0.0678
Interaction study*age	1, 201	0.60	0.4243	[0.4106, 0.4380]	0.0030
Interaction study*condition	2, 402	0.04	0.8182	[0.8075, 0.8289]	0.0010
Interaction age*condition	2, 402	0.04	0.9578	[0.9522, 0.9634]	0.0002
Interaction study*age*condition	2, 402	0.40	0.6609	[0.6477, 0.6740]	0.0020

Post hoc tests for main effect condition	df	*t*	*p	CI 95%	*d*
Positive vs neutral	204	−4.63	0.0001	[−0.0001, 0.0005]	−0.32
Negative vs neutral	204	−3.85	0.0001	[−0.0001, 0.0005]	−0.26
Positive vs negative	204	−0.89	0.3589	[0.3456, 0.3722]	−0.06

*Adjusted alpha α = 0.05/3 = 0.0167.

**Table 2:** Full model comparison for the Bayesian 2 × 2 × 3 mixed ANOVA.

Model	BF_10_
Null Model	1.0000
Condition	38217.93
Age	6.45 × 10^8^
Condition + age	2.44 × 10^13^
Condition + age + condition*age	1.01 × 10^12^
Study	0.31
Condition + study	12771.44
Age + study	1.81 × 10^8^
Condition + age + study	7.03 × 10^12^
Condition + age + condition*age + study	2.84 × 10^11^
Condition + study + condition*study	573.35
Condition + age + study + condition*study	2.94 × 10^11^
Condition + age + condition*age + study + condition*study	1.44 × 10^10^
Age + study + age*study	9.87 × 10^7^
Condition + age + study + age*study	3.51 × 10^12^
Condition + age + condition*age + study + age*study	1.17 × 10^11^
Condition + age + study + condition*study + age*study	1.73 × 10^11^
Condition + age + condition*age + study + condition*study + age*study	7.44 × 10^9^
Condition + age + condition*age + study + condition*study + age*study + condition*age*study	4.20 × 10^8^

All Bayes Factors reflect the comparison to the null model.

*BF* Bayes Factor.

### Explicit cue valence ratings

The explicit cue valence rating was part of the SID-Video study (Fig. 1b). Here, we measured subjective emotional cue valence by using a rating scale ranging from unpleasant to pleasant (coded as −10 and 10, respectively). To keep a similar structure for both SID tasks, we added extra blocks at the end of the SID-Video task including trials in which the cue was followed by the rating instead of the target.

**Fig. 4:**
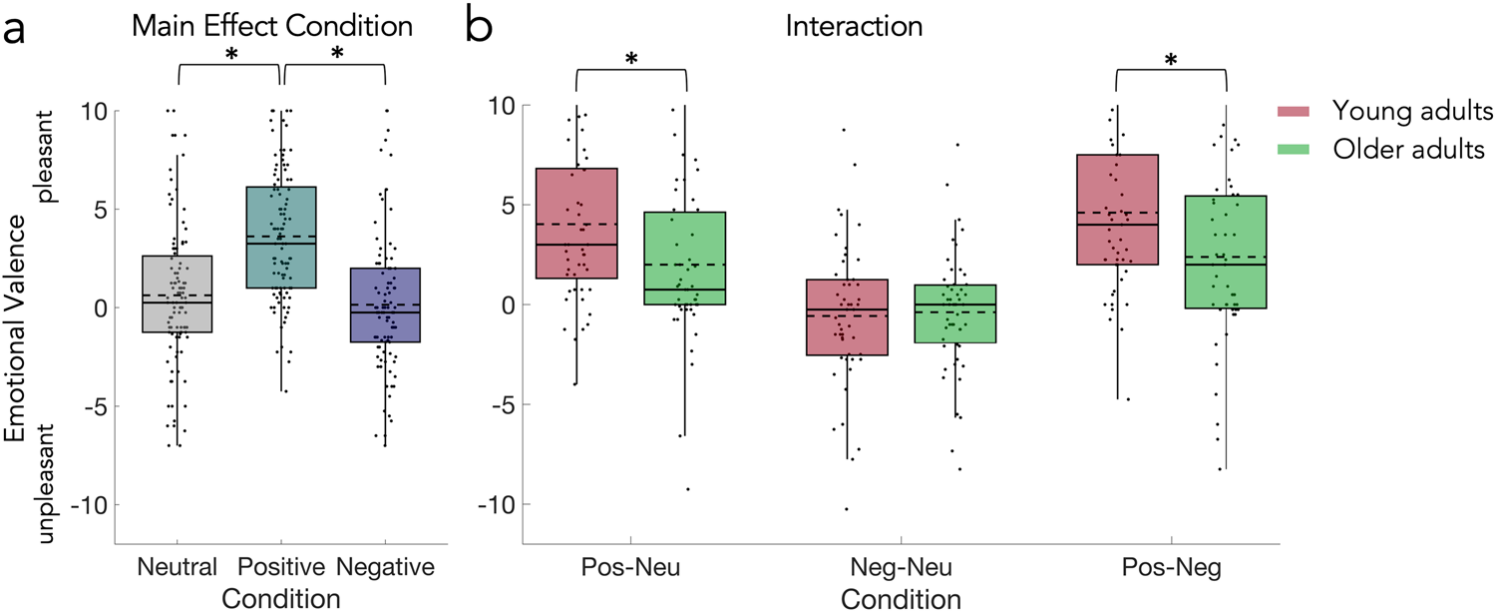
Results of the explicit cue valence rating. **a** Significant main effect of condition and significant differences between positive vs neutral and positive vs negative but not negative vs neutral condition. **b** Differences between conditions in emotional valence ratings for young and older adults. The rating differences between positive and neutral cue as well as positive and negative cue were significantly smaller in the older adults. Statistical comparisons were made using a 2 × 3 mixed design (between factor age; within-factor condition) permutation *F*-test and two-sided two-sample permutation *t*-tests (with Bonferroni correction). Data is presented in boxplots overlaid with data points. Boxes span from the lower to the upper quantile and whiskers of the boxplots depict data points that are the furthest from the center while still being inside the range of 1.5 times the interquartile range from the lower or upper quartile. Solid lines indicate the median, dashed lines indicate the mean. Asterisks mark significant differences (*p* < 0.0167).

On average, the valence of the neutral cue was rated 0.00 (SD = 3.38) by young and 1.21 (SD = 3.80) by older adults, while the valence of the positive cue was rated 4.03 (SD = 2.98) by young and 3.21 (SD = 3.60) by older adults, and the valence of the negative cue was rated −0.56 (SD = 3.06) by young and 0.83 (SD = 3.72) by older adults. The 2 × 3 mixed design permutation *F*-test (age × condition) showed a significant main effect of condition (*p* = 0.0001; Fig. 4a) and a significant interaction of the two factors (*p* = 0.0082; Fig. 4b), but no significant main effect of age. Post-hoc permutation paired *t*-tests for the main effect of condition (Fig. 4a) showed significant differences between positive vs neutral (*p* = 0.0001) and positive vs negative (*p* = 0.0001), but not negative vs neutral (*p* = 0.1428). The post-hoc two-sample permutation *t*-tests comparing the average ratings in each condition between young and older adults showed no significant effects. Therefore, we performed three additional exploratory post-hoc two-sample permutation *t*-test (Fig. 4b), which revealed that the differences between positive and neutral (*p* = 0.0106) as well as positive and negative (*p* = 0.0150) but not negative and neutral cue ratings are significantly larger in the young compared to the older adults. See for the statistical details.

### Implicit cue valence—SC-IAT

**Fig. 5:**
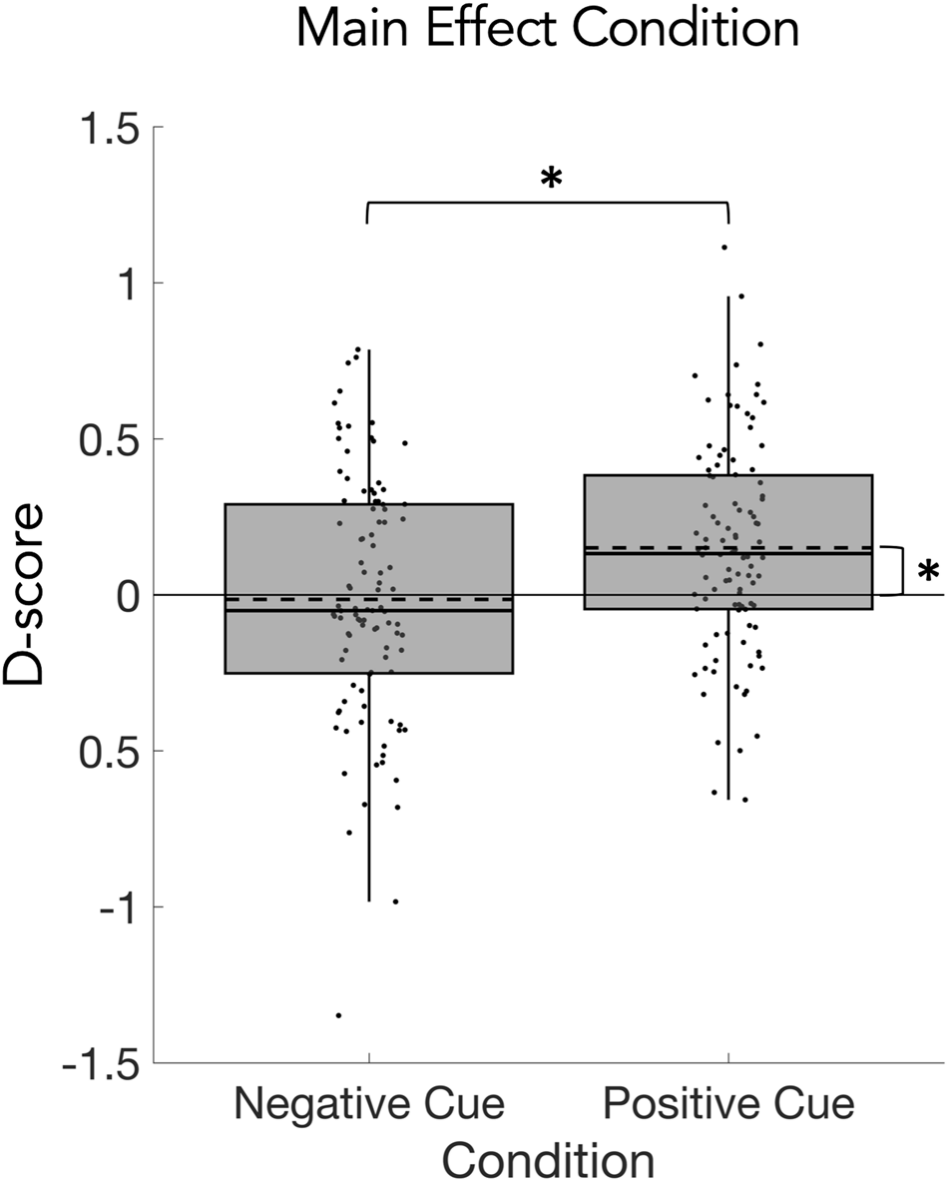
Results of the single category implicit association test. D-scores for the positive and negative cues averaged across young and older adults. The D-score designates an association of the cue with positive or negative valence. D-scores for the positive cue were significantly higher than for the negative cue (main effect of condition). Only the D-score for the positive cue was significantly different from zero. Statistical comparisons were made using a 2 × 2 mixed design (between factor age; within-factor condition) permutation *F*-test and two-sided one-sample permutation *t*-tests (with Bonferroni correction). Data is presented in boxplots overlaid with data points. Boxes span from the lower to the upper quantile and whiskers of the boxplots depict data points that are the furthest from the center while still being inside the range of 1.5 times the interquartile range from the lower or upper quartile. Solid lines indicate the median, dashed lines indicate the mean. Asterisks mark significant differences (*p* < 0.005).

The Single Category Implicit Association Test (SC-IAT)^36^ was used to assess implicit cue valence (Fig. 1c). It is based on the notion that processing highly associated categories leads to faster RTs^35^ despite being unaware of them. The strength of the association is assumed to be reflected in a D-score: positive D-scores indicate a positive valence association with the cue, while negative D-scores indicate a negative valence association. On average, the D-score for the positive cue was 0.14 (SD = 0.33) in young and 0.16 (SD = 0.34) in older adults while the D-score for the negative cue was 0.04 (SD = 0.37) in young and −0.06 (SD = 0.40) in older adults. The 2 × 2 mixed design permutation *F*-test (age x condition) on D-scores from the SC-IAT showed a significant main effect of condition (*p* = 0.0042; Fig. 5) but no significant main effect of age and no significant interaction. The one-sample permutation *t*-tests on the D-scores for the positive and negative cue across both age groups showed that the D-score for the positive cue was significantly different from zero (*p* = 0.0003) while the D-score for the negative cue was not (Fig. 5). See Table 3 for the detailed statistics.

**Table 3:** Results of the explicit cue valence ratings and Single-Category Implicit Association Test (SC-IAT) on implicit cue valence.

Explicit cue valence					
2 × 3 *F*-test	df	*F*	*p*	CI 95%	η2p
Main effect age	1, 98	1.32	0.2553	[0.2433, 0.2674]	0.0133
Main effect condition	2, 169	46.58	0.0001	[−0.0001, 0.0005]	0.3222
Interaction age*condition	2, 196	4.89	0.0082	[0.0057, 0.0107]	0.0476

Post hoc tests for main effect condition	df	*t*	*p	CI 95%	*d*
Positive vs neutral	99	7.14	0.0001	[−0.0001, 0.0005]	0.71
Negative vs neutral	99	−1.47	0.1428	[0.1331, 0.1525]	−0.14
Positive vs negative	99	7.79	0.0001	[−0.0001, 0.0005]	0.77

Post hoc tests for interaction	df	*t*	*p	CI 95%	*d*
Young vs older: Neutral	98	−1.68	0.1006	[0.0922, 0.1089]	−0.33
Young vs older: Positive	98	1.22	0.2200	[0.2085, 0.2314]	0.24
Young vs older: Negative	98	−2.04	0.0440	[0.0383, 0.0497]	−0.40

Exploratory post hoc tests	df	*t*	*p	CI 95%	*d*
Young vs older: Positive – neutral	99	2.47	0.0106	[0.0078, 0.0134]	0.49
Young vs older: Negative – neutral	99	−0.29	0.7818	[0.7704, 0.7933]	−0.05
Young vs older: Positive – negative	99	2.55	0.0150	[0.0116, 0.0184]	0.51

Implicit cue valence					
2 × 2 *F*-test	df	*F*	*p*	CI 95%	η2p
Main effect age	1, 98	0.78	0.3951	[0.3816, 0.4087]	0.0079
Main effect condition	1, 98	9.27	0.0042	[0.0024, 0.0060]	0.0864
Interaction age*condition	1, 98	1.44	0.2358	[0.2240, 0.2475]	0.0145

One-sample tests	df	*t*	**p		*d*
Positive	99	4.47	0.0003		0.45
Negative	99	−0.36	0.7121		−0.03

*Adjusted alpha α = 0.05/3 = 0.0167.

**Adjusted alpha α = 0.05/2 = 0.025.

### Emotional valence ratings of feedback videos

The emotional valence of the video feedback stimuli was rated on a scale from negative to positive (coded as −10 and 10, respectively) to assess age-related differences in socio-emotional processing. Although the video stimuli were validated in a pre-study (see methods for details), we also used the data to ensure significant differences in valance ratings and agreement with the intended emotional valence.

**Fig. 6:**
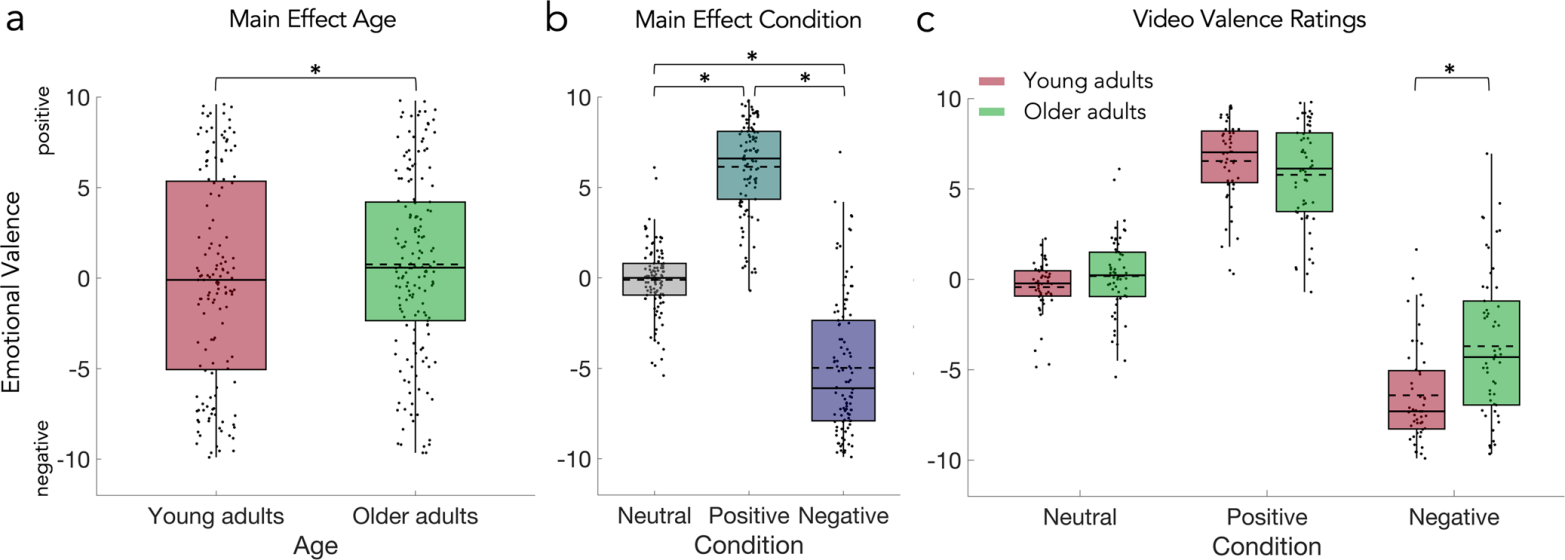
Results of the explicit emotional valence rating for the feedback videos. The analysis also showed (**a**) a significant main effect of age and (**b**) a significant main effect of condition. Ratings of neutral, positive, and negative feedback videos were significantly different from each other. The condition averages corresponded with the indented valence. **c** The analysis showed a significant interaction which resulted from the significantly less negative ratings of negative feedback videos by the older adults. Statistical comparisons were made using a 2 × 3 mixed design permutation *F*-test (between factor age; within-factor condition), two-sided permutation paired *t*-tests, and two-sided two-sample permutation *t*-tests (with Bonferroni correction). Data are presented in boxplots overlaid with data points. Boxes span from the lower to the upper quantile and whiskers of the boxplots depict data points that are the furthest from the center while still being inside the range of 1.5 times the interquartile range from the lower or upper quartile. Solid lines indicate the median, dashed lines indicate the mean. Asterisks mark significant differences (*p* < 0.001).

On average, the valence of neutral feedback videos was rated −0.43 (SD = 1.47) by young and 0.17 (SD = 2.21) by older adults, while valence of positive feedback videos was rated 6.54 (SD = 2.32) by young and 5.78 (SD = 2.80) by older adults; valence of negative feedback videos was rated −6.41 (SD = 2.75) by young and −3.69 (SD = 4.11) by older adults. The 2 × 3 mixed design permutation *F*-test (age x condition) on the emotional valence ratings of feedback videos showed a significant main effect of age (*p* = 0.0003; Fig. 6a), a significant main effect of condition (*p* = 0.0001; Fig. 6b), and a significant interaction (*p* = 0.0003; Fig. 6c). The post-hoc permutation paired *t*-tests (Fig. 6b) showed significant differences between the positive vs neutral (*p* = 0.0001) and negative vs neutral (*p* = 0.0001), and positive vs negative (*p* = 0.0001) condition. The post-hoc two-sample permutation *t*-tests for the interaction comparing the average ratings in each condition between the young and older adults showed a significant difference in the ratings of the negative feedback videos (*p* = 0.0003, Fig. 6c), but no differences between the ratings of young and older adults in the neutral and positive condition. See Table 4 for the complete statistics.

**Table 4:** Results of the emotional valence and credibility ratings for feedback videos.

Emotional valence					
2 × 3 *F*-test	df	*F*	*p*	CI 95%	η2p
Main effect age	1, 100	11.11	0.0003	[−0.0001, 0.0009]	0.1000
Main effect condition	2, 200	359.42	0.0001	[−0.0001, 0.0005]	0.7823
Interaction age*condition	2, 200	8.71	0.0003	[−0.0001, 0.0009]	0.0802

Post hoc tests for main effect condition	df	*t*	*p	CI 95%	*d*
Positive vs neutral	101	18.26	0.0001	[−0.0001, 0.0005]	1.80
Negative vs neutral	101	−14.49	0.0001	[−0.0001, 0.0005]	1.43
Positive vs negative	101	19.16	0.0001	[−0.0001, 0.0005]	1.89

Post hoc tests for interaction	df	*t*	*p	CI 95%	*d*
Young vs older: Neutral	100	−1.61	0.1102	[0.1015, 0.1189]	−0.32
Young vs older: Positive	100	1.48	0.1384	[0.1288, 0.1479]	0.29
Young vs older: Negative	100	−3.86	0.0003	[−0.0001, 0.0009]	−0.76

Credibility					
2 × 3 *F*-test	df	*F*	*p*	CI 95%	η2p
Main effect age	1, 102	2.52	0.1140	[0.1050, 0.1226]	0.0242
Main effect condition	2, 204	0.35	0.6911	[0.6783, 0.7039]	0.0035
Interaction age*condition	2, 204	22.23	0.0001	[−0.0001, 0.0005]	0.1790

Post hoc tests for interaction	df	*t*	*p	CI 95%	*d*
Young vs older: Neutral	102	2.87	0.0050	[0.0030, 0.0070]	0.56
Young vs older: Positive	102	−4.46	0.0001	[−0.0001, 0.0005]	−0.87
Young vs older: Negative	102	−1.49	0.1316	[0.1222, 0.1409]	−0.29

*Adjusted alpha α = 0.05/3 = 0.0167.

### Credibility ratings of feedback videos

Participants were instructed to report the credibility of the feedback and emotion in the video clips on a scale from unbelievable to believable (coded as −10 and 10, respectively). Although the video stimuli were validated in a pre-study (see methods for details), we also used the data to check for systematic differences that might influence the interpretation of other results.

**Fig. 7:**
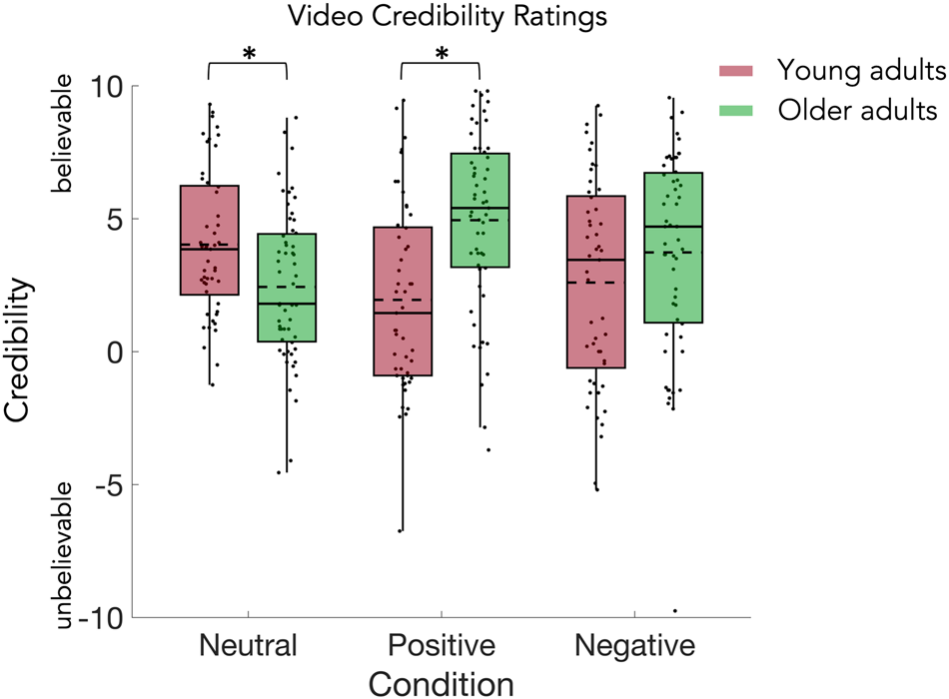
Results of the credibility rating for the feedback videos. On average, the credibility of the feedback videos was positive. Ratings by young and older adults differed significantly in the neutral and positive condition. Statistical comparisons were made using a 2 × 3 mixed design permutation *F*-test (between factor age; within-factor condition) and two-sided two-sample permutation *t*-tests (with Bonferroni correction). Data are presented in boxplots overlaid with data points. Boxes span from the lower to the upper quantile and whiskers of the boxplots depict data points that are the furthest from the center while still being inside the range of 1.5 times the interquartile range from the lower or upper quartile. Solid lines indicate the median, dashed lines indicate the mean. Asterisks mark significant differences (*p* < 0.01).

On average, the credibility of neutral feedback videos was rated 4.02 (SD = 2.73) by young and 2.43 (SD = 2.88) by older adults, while credibility of positive feedback videos was rated 1.94 (SD = 3.53) by young and 4.94 (SD = 3.31) by older adults; credibility of negative feedback videos was rated 2.59 (SD = 3.91) by young and 3.73 (SD = 3.79) by older adults. The 2 × 3 mixed design permutation *F*-test (age x condition, Fig. 7) on the credibility ratings of feedback videos showed a significant interaction (*p* = 0.0001), but no significant main effect of age and no significant main effect of condition. The post-hoc two-sample *t*-tests for the interaction, comparing the average ratings in each condition between the young and older adults, showed significant differences in the neutral (*p* = 0.0050; Fig. 7), and positive (*p* = 0.0001; Fig. 7), but not negative condition. See Table 4 for the detailed statistics.

### Partial correlations of explicit and implicit cue valence with feedback valence ratings

**Fig. 8:**
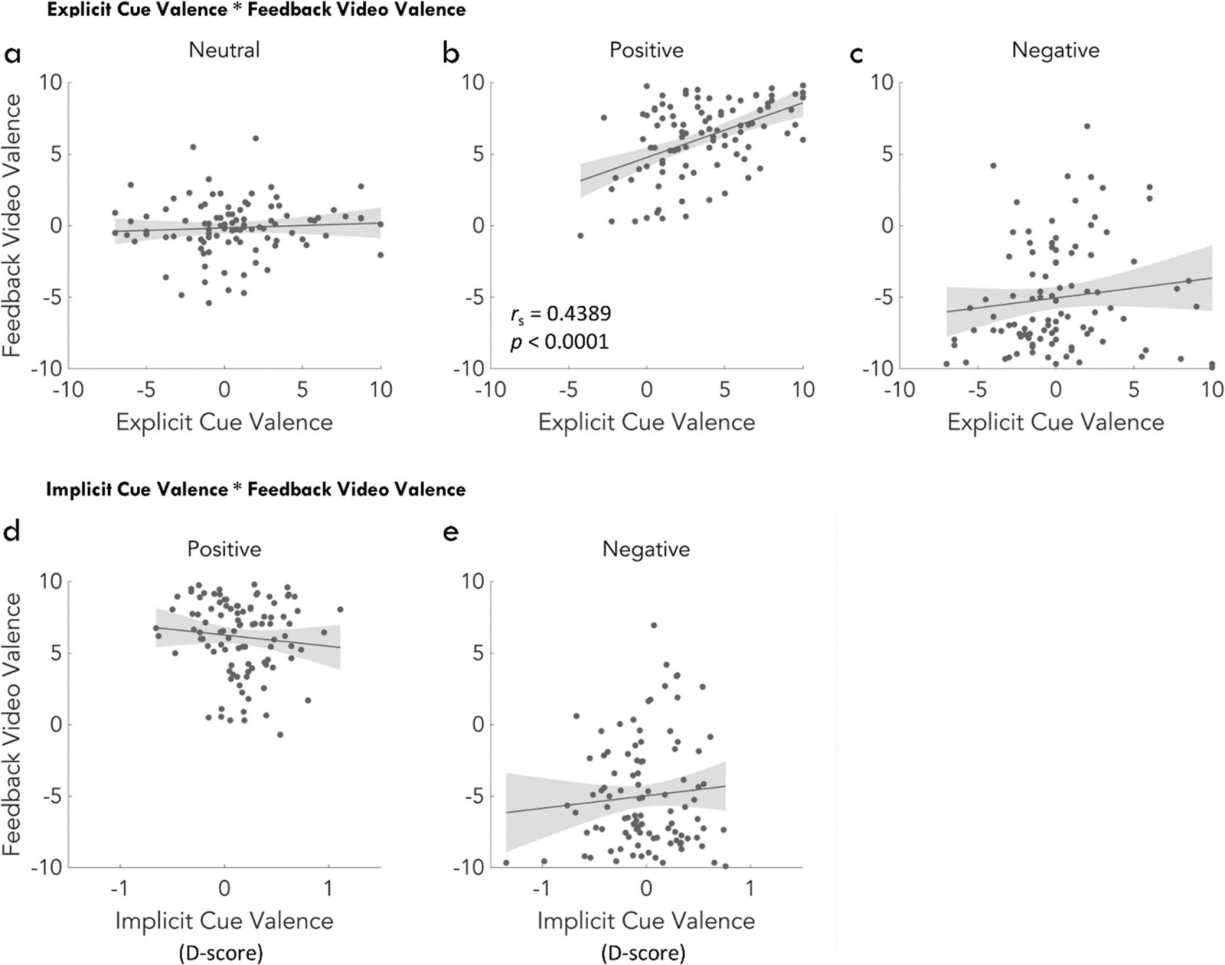
Results of the partial correlations between cue valence and feedback valence. **a**–**c** Partial correlations between explicit cue valence and feedback video valence in the neutral, positive, and negative condition. Cue valence and feedback video valence were significantly correlated in the positive condition. **d**, **e** Partial correlations between implicit cue valence and feedback video valence in the positive and negative condition. Statistical analyses were made condition using Spearman correlation coefficients (two-sided, with Bonferroni correction). Plots show the individual data points together with the fitted generalized linear model (GLM) and 95% confidence bounds (shaded area).

To further investigate evaluative learning, we assessed the link between cue valence and feedback video valence by using partial correlations controlling for age. Explicit cue valence correlated with feedback video valence in the positive condition (r_s_ = 0.4389, *p* < 0.0001; Fig. 8b) but not in the neutral (r_s_ = 0.0596, *p* = 0.5597; Fig. 8a) or negative (r_s_ = 0.0856, *p* = 0.4018; Fig. 8c) condition. Implicit cue valence did not correlate with feedback video valence in any condition (positive: r_s_ = −0.1302, *p* = 0.2012; Fig. 8d; negative: r_s_ = 0.1144, *p* = 0.2619; Fig. 8e).

## Discussion

We investigated the effects of more realistic social feedback on motivation, evaluative learning, and socio-emotional processing in young (18–35 years) and older (50–84 years) adults. Overall, RT was accelerated by the possibility to obtain positive and avoid negative social feedback regardless of age, which confirms previous work. More realistic dynamic video feedback did not further accelerate RT but valence ratings of video feedback stimuli showed age-related differences that are compatible with a typical age-related positivity bias in socio-emotional processing. Regarding evaluative learning, explicit and implicit measures of emotional cue valence revealed that reward cues were associated with positive, and punishment cues with neutral emotional valence. Explicit cue valence correlated with feedback video valence in the positive condition independent of age, indicating a specific transfer of emotional valance. As such, our results suggest that dynamic and static social feedback act as motivators across the life span. Further, older and younger adults alike show evaluative learning for predictors of positive social feedback but the socio-emotional processing of dynamic social stimuli is modulated by age.

Human behavior, including learning and motivation, is profoundly modulated by social reinforcers. Compatible with such a view and the existing literature^8,10^, we find that RTs are faster when anticipating positive and negative compared to neutral social feedback in both young and older adults (Fig. 3). At the neural level, a behavioral advantage by social and monetary reinforcers has often been linked to the mesolimbic system, including the dopaminergic midbrain, ventral striatum and other interconnected brain regions, such as the prefrontal cortex^27,37^. Importantly, the mesolimbic system degenerates during healthy aging^25^ leading to impaired learning and memory^23,26^. However, the absence of age-related differences in RT acceleration, as observed here, is in line with the notion that social affiliation constitutes a fundamental human motive across the life span^33^, and they could also be accounted for by the simple nature of our task. Indeed, age-related impairments are often reported in more difficult learning tasks^38^ and they are probably based on reduced prediction error signaling in frontostriatal circuits^23^. Moreover, the restoration of the prediction error via L-DOPA administration^39^ and providing additional information about future values^40^ improved learning in older adults. Our results are consistent with these observations and indicate that possible age-related declines of the mesolimbic system do not necessarily affect RT in the SID task. In a broader context, our observations further underline that older adults are capable of behavioral adaptation and learning under specific circumstances.

The finding that RTs accelerated for static photo and dynamic video feedback alike (Fig. 3) was unexpected given that dynamic social information should be more immersive and therefore promote behavioral invigoration^8,16^. Instead, our results suggest that behavioral invigoration (as indicated by RT acceleration) is normalized to the distribution of available options within a context. This view is compatible with the “value normalization framework”, which was originally proposed for neural responses to rewards in choice tasks^41^. However, it may also be applicable to other settings^42^, and therefore useful to explain the behavioral effects in our study. Precisely, the presentation of static photo and dynamic video feedback in two separate tasks (instead of a trial-by-trial variation) may have led to normalized rank values of positive and negative social feedback relative to neutral feedback within each experimental context. As a result, RTs were equally accelerated to obtain positive and avoid negative social feedback regardless of static or dynamic feedback. A similar rationale has been proposed in the context of “adaptive scaling” of reward predictions errors, as shown in monkeys^43^ and the human mesolimbic system^44^. Together, anticipating positive and negative social feedback accelerated RTs in young and older adults which indicates increased motivation, but there was no additional benefit of using dynamic video feedback. This result may be explained by context dependent value normalization and may translate to other measures of physical effort. Nonetheless, more research is needed to test this hypothesis and the effects of increasingly naturalistic social stimuli^45^ on motivation and cognition.

Our older adults had less pronounced ratings of negative social feedback videos (Fig. 6), which is compatible with the literature on the age-related positivity effect typically shown for static pictures such as faces^5,30,31^. While this has often been interpreted as prioritization of emotional goals in older adults^33^, we can extend the literature by demonstrating the positivity effect in a set of more ecologically valid affective social video stimuli. From a more mechanistical point of view, the positivity effect in older adults may be an expression of cognitive change strategies, specifically reappraisal^46^, used for emotion regulation. These strategies can help to achieve emotional goals^47^ depending on specific contexts. In our study, the reappraisal of social stimuli in older adults did not reduce the motivation to avoid negative feedback but seems to be limited to the exposition to negative emotional content. This may be, again, explained by value normalization or adaptive scaling, or the primacy of early emotion regulation in younger and older adults^48^, such as the avoidance of negative information^46^. Furthermore, according to the strength and vulnerability integration model, older adults have difficulties in recovering from sustained arousal^49^ leading to the avoidance of negative situations. Together, while young and older adults differ in their socio-emotional processing when being confronted with negative affective social information, they are similarly motivated to avoid negative social information when possible.

We found evidence for evaluative learning of cue valence in young and older adults. Specifically, our data indicate the transfer of positive emotional valence from dynamic social feedback to the initially neutral cues (Figs. 4 and 5). Moreover, explicit valence ratings of positive cues correlated with valence ratings of positive feedback videos (Fig. 8). In general terms, these results are compatible with the notion of evaluative learning and valence transfer^21,22^ and they demonstrate that older adults are still able to learn emotional associations from positive dynamic social feedback for predictive cues, while age-related problems in implicit learning may occur in a task-dependent manner, for example in probabilistic sequential learning settings^26^. In contrast, ratings and D-scores for the negative cues were not significantly different from neutral (Figs. 4 and 5) and no correlation was found between negative cues and negative feedback videos (Fig. 8). While this indicates a specific effect for positive cues and feedback, it may be explained by the different numbers in neutral, positive, and negative feedbacks. In fact, positive cues preceded positive feedback in 83% of the trials but negative cues preceded negative feedback only in 17% of the trials. While this imbalance is a limitation and higher rates of negative feedback could have induced evaluative learning for the negative cues, we intended to not frustrate or demotivate our participants.

The assessment of cue valence was characterized by age effects in the explicit (Fig. 4) but not in the implicit test (Fig. 5). Specifically, for explicit ratings of cue valence, the differences of positive vs neutral and positive vs negative were smaller in older compared to younger adults. On the one hand, this could imply weaker explicit emotional memory in older adults along with general deficits in explicit memory^50^. On the other hand, these results could be interpreted in a dual-process framework of cognition^51^. From this perspective, the explicit cue and video ratings are influenced by higher order cognitive processes, e.g., emotion regulation strategies, while the results of implicit measures reflect automatic responses. This interpretation is further supported by a significant correlation of explicit but not implicit cue ratings with feedback video ratings in the positive condition.

The actors in the feedback videos were 24 and 27 years old and may have been perceived differently by the two age groups. This is also known as intergroup biases^52^, which could directly or indirectly affect learning, socio-emotional processing, and motivation. In fact, faces of the own age group are better remembered than faces of other age groups^53,54^ and higher amounts of contact to the own group reduces emotion recognition in the other age group^53^. However, both, young and older participants, are better at recognizing emotions from young than older faces^53^ probably due to stronger expressivity in younger faces^55^. Moreover, emotions induced by receiving positive social feedback^56^ or viewing emotional faces^57^ are not modulated by the source (ingroup vs outgroup). In addition, attitudes towards specific age groups could influence how the feedback is perceived, for example depending on the competence attributed to the person giving feedback. However, while younger adults (21–35 years) favored the ingroup by devaluating the competence of middle-aged (36–54 years) and older (>55 years) adults, older adults showed no bias in favor of their own age group^58^. Regarding motivation, individuals are more willing to exert efforts to enhance the impression that other ingroup members have compared to the outgroup^56^. Together, older adults typically show no own age bias in emotion recognition or competence attribution, and emotional responses to feedback are typically not modulated by ingroup vs outgroup settings. However, intergroup biases in the motivation to gain social approval from the ingroup compared to the outgroup could have an influence although this was not explicitly shown for different age groups.

Another aspect that should be noted is that photo and video feedback stimuli differed not only with respect to dynamics but also durations (750 ms and 2400 ms, respectively). From a conceptual point of view, photo and video duration should have been matched, e.g., by a longer presentation of the photos. However, from a motivational point of view, prolonged static image presentation could induce boredom, which is an unpleasant emotional state leading to less task engagement and motivation.

Finally, we would like to discuss possible limitations of our study design, i.e., between vs within subjects. The exposure to multiple kinds of incentives within subjects has psychological consequences and the valuation of static and dynamic social incentives may depend on the availability of the other^59^. To this end, it is unclear whether a within-subjects design would have produced the same or different results, and our study is informative for future studies and possible learning tools, which most likely would implement one but not both types of social feedback. From a practical point of view, having both conditions (static and dynamic feedback) with similar trial numbers as we have in our current version, a possible experiment would be twice as long. This could have a negative effect on motivation and behavior and, therefore, the validity of the results. One disadvantage of using a between-subject design are context effects. Specifically, the SID-Video study was performed during the COVID-19 pandemic while the SID-Photo study was conducted one year before. Due to social distancing and contact restrictions, the social interaction with the experimenters might have increased the subjects’ motivation, leading to greater effort and, therefore, to overall faster RTs than in the SID-Photo study. Thus, differences in RTs between studies could be coincidental, and the effects of realistic social feedback on motivation may have been masked by the aforementioned effects.

To conclude, anticipating positive and negative social feedback is an essential part in the learning process that invigorates behavior in young and older adults, but there was no additional benefit of dynamic over static social stimuli. Our data also indicate a transfer of emotional valence from social video feedback to initially neutral cues in both young and older subjects, which differed between groups only in the explicit task. Together with a positivity effect in older adults, this is compatible with theories on dual-processing as well as cognitive control strategies for emotion regulation and it indicates specific developmental changes in evaluative learning during healthy aging. As such, our work gives insights into the effects of age on motivation, learning, and socio-emotional information processing.

## Methods

### Inclusion and exclusion criteria

All participants were screened for pre-established inclusion and exclusion criteria prior to the experiment. Participants were required to be currently healthy, of adult age (18–35 years, or >50 years), contractually capable, right-handed, fluent in German, not consume any illegal drugs, consume less than 15 glasses of alcohol per week, and less than 15 cigarettes per day. Exclusion criteria were defined as previous or current medical conditions including psychiatric illnesses, mild cognitive impairment (MCI), dementia, heart conditions, cardiovascular diseases, or other severe illnesses (in particular those that affect the central nervous system), current medical treatment up to 2 weeks before the study excluding prescription-free medications and oral contraceptives.

### Participants

In this study, we combined new and previously recorded and published behavioral data^9^ to compare the SID task using photo vs video stimuli in young and older adults. All older adults were screened for MCI and dementia using the Montreal Cognitive Assessment^60^ (MoCA; scores <22 indicate MCI or dementia^61^). One young and three older participants of the SID-Video study were excluded due to fulfilling pre-established exclusion criteria (the young participant reported depression in clinical history, one older participant reported a stroke in clinical history and two older participants scored below 22 in the MoCA). We included 52 young (age range = 18–32; M ± SD = 22.8 ± 3.1 years; *n* female = 29) and 52 older adults from the SID-Photo study (age range = 51–75; M ± SD = 64.0 ± 6.4 years; *n* female = 29) and another 49 young (age range = 18–35; M ± SD = 23.9 ± 3.7 years ; *n* female = 36) and 55 older adults for the SID-Video study (age range = 50–84; M ± SD = 64.2 ± 8.2 years; *n* female = 31; MoCA mean score = 27.9; SD = 1.5; all MoCA scores ≥22). Final sample sizes for each task (see statistical analyses) can vary due to technical problems in data acquisition and will therefore be reported separately for each task. This study was approved by the local ethics committee of the University of Lübeck, Germany. All participants gave written informed consent, in accordance with the Declaration of Helsinki, before taking part in the study.

### Recruitment and testing procedure

We recruited our participants for both studies via student mailing lists, newspapers, public spaces, and the department’s database^62^. The younger group mainly includes students from the University of Lübeck and Technische Hochschule Lübeck, while the older group mainly consists of volunteers from Lübeck and its greater catchment area. For the SID-Photo study, data for the young sample was collected from March 2016 until May 2016 and data for the older was collected from February 2019 until April 2019. The data collection for the SID-Video study (young and older sample) was initially planned to start in April 2020 but it was postponed due to the COVID-19 pandemic. Data for the SID-Video study was collected from October 2020 until June 2021—a time of social distancing and contact restriction measures. Specifically, the data collection period for the SID-Video study included partial and full lockdowns from November 2020 until March 2021.

Data collection in the SID-Photo study was carried out by two female experimenters. One collected data for the young participants and the other for the older participants. Data collection for the SID-Video study was carried out by four other female experimenters who collected data for both young and older participants. Importantly, all experimenters followed a standardized instruction protocol to avoid potential biases. The experiments were performed in a lab environment and the participant performed the tasks in a separate test room while the experimenter was in a control room.

Both studies used similar laptops to perform the tasks. The SID-Photo study was programmed using Cogent Graphics developed by John Romaya at the Laboratory of Neurobiology at the Wellcome Department of Imaging Neuroscience. The SID-Video study was programmed in MATLAB 2018b, using the Psychophysics Toolbox extensions (Version 3)^63,64^ since video playback was not possible using Cogent.

The SID-Photo study included three parts: (1) a simple RT task, (2) the SID Photo task (preceded by a short training), and (3) a rating task assessing emotional valence of the feedback videos. The SID-Video study included five parts: (1) a simple RT task, (2) the SID Video task (preceded by a short training), (3) an explicit rating task assessing explicit cue valence, (4) a version of the Single Category Implicit Association Test^36^ assessing implicit cue associations, and (5) a rating task assessing emotional valence and credibility of the feedback videos. Note that the emotional valence rating in the SID-Photo study was designed differently^9,65^ and was not reused or reanalyzed as part of this study.

### Paradigm

The simple RT task comprised 50 trials and was subsequently used in a staircase scenario of the SID task as described in our previous publication^9^. The SID task used the same trial structure and timings as in our previous publication^9^ and only differed with regard to the feedback stimuli and their timing (Fig. 1a). Photos were presented for 750 ms and videos had an average duration of 2400 ms (SD = 400 ms). In brief, we presented a cue, followed by a target (green circle), followed by the feedback. Participants had to respond to the target as fast as possible via button press (keyboard or mouse were used). The cues signaled condition (neutral, positive, negative) and potential feedback to be received. In the positive condition, positive feedback was received for fast responses and in the negative condition, negative feedback was received for slow responses, in the neutral condition the feedback was always neutral. Here, we used videos of one female and one male volunteer with acting experience showing neutral, positive, and negative mimics (e.g., neutral, smiling, angry), gestures (e.g., thumbs horizontal, thumbs up, thumbs down, but also other hand movements), and verbal feedback (e.g., “Quite okay.”, “Well done!”, “That was not good.”). We varied hand movements, verbal feedback, and mimic details to make the feedback more realistic. Each condition comprised 16 different videos (8 female, 8 male) as feedback. A pool of 12 videos (4 per condition) were used in the short training. The SID comprised six blocks (2 per condition) in a randomized order. Note that social feedback in the SID Photo task was provided by 10 different volunteers (5 female, 5 male). Each condition comprised 10 photographs as feedback, one of each volunteer. On these photographs, the volunteers showed neutral, positive or negative mimics (i.e., neutral, smiling, angry) and gestures (i.e., right thumb horizontally, right thumb up, right thumb down).

The explicit rating task, assessing explicit cue valence, was implemented by appending the SID task by one extra block per condition including four extra trials each. In these trials, the cue was followed by a screen instead of the target, asking the participants to rate their emotion about the previously presented cue ranging from unpleasant to pleasant. The rating was made on a line with two end points (coded as −10 and 10, respectively) and a dot in the middle that could be moved by pressing the arrow keys on the keyboard (Fig. 1b).

The Single Category Implicit Association Test (SC-IAT) was used to assess implicit cue valence. It is a modification of the implicit association test^35^ and was adapted according to Karpinski and Steinman^36^. Here, we presented four experimental blocks (60 trials each; positive congruent, positive incongruent, negative congruent, and negative incongruent) each preceded by a short training block (20 trials). In each trial, a target stimulus appeared in the middle of the screen and the participants had to respond by pressing either “x” or “m” on a keyboard. Depending on the block, the target stimuli were words with positive and negative valence and the respective positive or negative cue from the previous SID task (Fig. 1c). In the positive congruent block, we presented 17 pleasant words, 26 unpleasant words, and 17 cues as targets; the positive incongruent block comprised 26 pleasant words, 17 unpleasant words, and 17 cues as targets; the negative congruent block comprised 26 pleasant words, 17 unpleasant words, and 17 cues as targets; the negative incongruent block comprised 17 pleasant words, 26 unpleasant words, and 17 cues as targets. The pleasant and unpleasant words were selected from the Berlin Affective Word List Reloaded (BAWL-R)^66^. Each block was preceded by a detailed instruction regarding the categorization and corresponding key responses. The participants always had to press the x-key for unpleasant words and the m-key for pleasant words. Additionally, in the positive congruent block the m-key had to be pressed for the previously positive cue while in the positive incongruent block the x-key was pressed for the previously positive cue. In the negative congruent block, the x-key had to be pressed for the previously negative cue and in the negative incongruent block the m-key had to be pressed for the previously negative cue. Generally speaking, congruency means that the valence that was signaled by the cue in the SID task (positive or negative cue) matches the valence of the assigned response key (i.e., press x-key for unpleasant words and negative cue) which was achieved by always pressing “x” for unpleasant words and “m” for pleasant words. Blocks in which the valence of the cue did not match the valence of the response key (i.e., press x-key for unpleasant words and positive cue) are labeled as incongruent trials. After a key press, participants received a green O as feedback for correct responses or a red X for incorrect responses. We prepared four versions of the SC-IAT with varying block order to prevent ordering effects and randomly select one of these versions for each participant.

A general idea of the IAT is that processing highly associated categories (i.e., congruent information) is associated with faster RTs^35^. Hence, if participants developed an association between a cue and emotional valence of the subsequent feedback, RTs in congruent blocks should be faster than in incongruent blocks.

The last part of the experiment aimed to evaluate the video feedback stimuli by presenting them to the participants, who were asked to rate the emotional valence and credibility (in the sense of authenticity and naturality). The order was randomized and each video was presented twice. The emotional valence was rated from negative to positive on a line with two end points (coded as −10 and 10, respectively) and a dot in the middle that could be moved by pressing the arrow keys on the keyboard. Credibility was rated from unbelievable to believable using the same scale design as for the valence rating.

### Creation and selection of video feedback stimuli material

We recorded a new set of social video feedback stimuli with one female (27 years old) and one male volunteer (24 years old) with acting experience. In total, we generated a set of 120 videos, 60 per volunteer matched for mimics, gesture, and verbal feedback (called matched video pairs in the following).

The videos were created to match the photos to a certain degree. For example, the gestures in the photos showed one thumb up, horizontal, or down. Therefore, we recorded the videos to show one thumb up, horizontal, or down, but additionally both thumbs up, horizontal, or down, as well as other gestures, e.g., a fist pump as a celebratory gesture in the positive feedback condition, to make the feedback appear more realistic, varied, and less like a playback. Moreover, all volunteers were Caucasian and wore white t-shirts in front of a gray background.

All videos were evaluated in an online study with *n* = 76 participants reporting fluent German reading and speaking skills (age range = 16–65 years, M ± SD = 27.3 ± 10.3, *n* female = 42, *n* male = 32, *n* diverse = 2) using lab.js^67^ and JATOS^68^ for rating their emotional valence and credibility. Further, we assessed the sympathy of the two volunteers, which was rated 2.6 for the female and 4.6 for the male actor (on a scale ranging from −10 to 10). The final selection process for the SID-Video task was based on a series of criteria: We selected videos pairs with a difference smaller than 1.5 in the average valence and credibility ratings. We then selected videos with an average credibility above 0 on a scale from −10 to 10. The remaining videos were selected based on matching gestures (showing two thumbs vs one thumb vs other gesture) across conditions. Finally, five matched video pairs (10 videos in total) per condition were selected for the SID-Video study. One showed one thumb up, horizontal, or down, and four showed both thumbs up, horizontal, or down. See Table 5 for an overview of the average valence and credibility ratings.

**Table 5:** Average ratings of emotional valence and credibility for the selected videos.

	Emotional Valence			Credibility		
	Neutral	Positive	Negative	Neutral	Positive	Negative
Female volunteer	−0.2	5.9	−6.2	1.0	2.1	1.3
Male volunteer	0.0	5.2	−5.1	1.4	2.1	2.0
Average	−0.1	5.5	−5.7	1.2	2.1	1.7

### Statistical analyses

All permutation tests described in this study were computed in MATLAB 2018b^69^ using built-in functions, custom scripts, and MATLAB code retrieved from File Exchange^70^. The permutation tests were computed as followed: first, empirical test statistics of interest (*F*-values or *t*-values) were obtained for the original sample. Second, to create the null condition, factor labels were permuted. Between-factor labels were permuted between subjects, while within-factor labels were permuted within subjects, keeping measurements together that belonged to the same subject. Third, the test statistics were computed for the permutation samples. In each analysis, 5000 permutation samples and corresponding test statistics were obtained to generate the permutation distribution. Fourth, it was assessed whether the observed empirical test statistic was unusually large for the distribution of permutation test statistics using a Monte Carlo *p*-value. Cohen’s *d* was calculated using MATLAB code retrieved from File Exchange^71^.

#### Simple RT task

We compared RT from the simple RT task between the SID-Photo and SID-Video study. First, for each individual data set, we eliminated trials with improbably short (<150 ms and <1st quartile – 1.5 * inter quartile range) and improbably long RTs (>3rd quartile + 1.5 * inter quartile range). We calculated the condition averages for each participant and conducted an outlier analysis using the Tukey method^72^. We removed one outlier in the group of older adults from the SID-Photo study and one from the SID-Video study resulting in *n* = 52 young and *n* = 51 older adults for the SID-Photo study, and *n* = 49 young and *n* = 54 older adults for the SID-Video study. We computed a 2 × 2 design (study x age) permutation *F*-test.

#### RT in SID-Photo and SID-Video

To evaluate the effect of photos vs videos as feedback on RT, we processed each participant’s RT data as described above and calculated condition averages for each participant. Then, we z-transformed the data sets from each study to account for baseline differences in RT between studies (see the results of the simple RT task), which implied that we could not evaluate the main effect of feedback type (study) on raw RTs but only interpret the relative differences between anticipating incentivized (i.e., positive and negative) and neutral feedback between static vs dynamic feedback. We conducted an outlier analysis using the Tukey method^72^ and eliminated one outlier from the group of young adults in the SID-Photo study and two from the group of young adults in the SID-Video study resulting in *n* = 51 young and *n* = 52 older adults for the SID-Photo study, and *n* = 47 young and *n* = 55 older adults for the SID-Video study.

We analyzed RT in a 2 × 2 × 3 mixed design (between factors study and age; within-factor condition with three levels: neutral, positive, negative) permutation *F*-test. Pair-wise comparisons between condition levels were computed using two-sided permutation paired *t*-tests (positive vs neutral, negative vs neutral, and positive vs negative) with an adjusted alpha-level controlling for multiple comparisons using Bonferroni correction (α = 0.05/3 = 0.0167). Because the expected interaction of the factors study and condition was not significant, we conducted a 2 × 2 × 3 mixed Bayesian ANOVA in jamovi (Version 1.6.23)^73^ using the default settings, to verify the results of the permuted mixed *F*-test and evaluate the evidence against the interaction (calculated as age + condition BF_10_/age + condition + age * condition BF_10_).

#### Explicit cue valence ratings

To analyze the scores from the explicit rating task in the SID-Video study, we computed the average of the four ratings in each condition (neutral, positive, negative) for each subject. Four older participants were removed from the analysis due to data loss resulting from technical problems. The data set contained no outliers as confirmed by an outlier analysis using the Tukey method^72^ leaving *n* = 49 young and *n* = 51 older adults for the analysis.

The explicit rating scores were analyzed using a 2 × 3 mixed design (between factor age; within-factor condition with three levels: neutral, positive, negative) permutation *F*-test. The significant main effect of condition was followed up by pair-wise comparisons using permutation paired *t*-tests (positive vs neutral, negative vs neutral, and positive vs negative; two-sided; α = 0.05/3 = 0.0167). To explore the significant interaction, we compared the average ratings in each condition between the young and older adults using three two-sample permutation *t*-tests with adjusted alpha-levels controlling for multiple comparisons using Bonferroni correction (two-sided; α = 0.05/3 = 0.0167). Since none of these tests were significant and would leave the interaction inconclusive, we conducted three exploratory post-hoc two-sample permutation *t*-test comparing the condition differences (positive minus neutral, negative minus neutral, and positive minus negative) between young and older adults with adjusted alpha-levels controlling for multiple comparisons using Bonferroni correction (two-sided; α = 0.05/3 = 0.0167).

#### Implicit cue valence—SC-IAT

Based on the SC-IAT and a suggestion by Karpinski and Steinman^36^, we calculated the so-called D-score, which designates an association of the cue with positive or negative valence. To this end, we first removed responses below 350 ms as well as above 1500 ms and replaced the RTs of incorrect responses by the block mean plus an error penalty of 400 ms. Then, we computed the average RTs for the positive congruent, positive incongruent, negative congruent, and negative incongruent blocks for each participant. To compute the individual D-scores for the positive cues, we subtracted the average RT of the positive congruent block from the average RT of the positive incongruent block. The resulting value was divided by the standard deviation of all RTs of correct responses in the positive congruent and positive incongruent block. For the negative cue, the D-scores were computed by subtracting the average RT of the negative incongruent block from the RT of the negative congruent block. The resulting value was divided by the standard deviation of all RTs of correct responses in the negative congruent and negative incongruent block. Positive D-scores designate associating the cue with positive valence while negative D-scores designate associating the cue with negative valence.

Two older participants were not included in the analysis due to data loss resulting from technical problems.

Error rates were below 20% in all participants. One young and one older adult were removed from data analyses due to extremely slow responses (>20% slower than 1500 ms). The data set contained no outliers as confirmed by an outlier analysis using the Tukey method^72^ leaving *n* = 48 young and *n* = 52 older adults for the analysis.

D-scores were analyzed using a 2 × 2 mixed design (between factor age; within-factor condition with two levels: positive, negative) permutation *F*-test. We further performed one-sample permutation *t*-tests on the D-scores for the positive and negative cue across both age groups with adjusted alpha-levels controlling for multiple comparisons using Bonferroni correction (two-sided; α = 0.05/2 = 0.025).

#### Emotional valence ratings of feedback videos

To evaluate the perceived emotional valence of feedback videos, we averaged the two ratings per video and then across all videos of one condition for each participant. We conducted an outlier analysis using the Tukey method^72^ and removed data from one outlier in the young adults and one in the older adults leaving *n* = 48 young and *n* = 54 older adults for the analysis. We analyzed the emotional valence ratings using a 2 × 3 mixed design permutation *F*-test (between factor age; within-factor condition with three levels: neutral, positive, negative). The significant main effect of condition was followed up by three pair-wise comparisons using permutation paired *t*-tests (positive vs neutral, negative vs neutral, and positive vs negative; two-sided) with adjusted alpha-levels controlling for multiple comparisons using Bonferroni correction (α = 0.05/3 = 0.0167). The significant interaction was explored by comparing the average ratings in each condition between young and older adults using three two-sample permutation *t*-tests with adjusted alpha-levels controlling for multiple comparisons using Bonferroni correction (two-sided; α = 0.05/3 = 0.0167).

#### Credibility ratings of feedback videos

The credibility ratings of feedback videos were first averaged per video and then across all videos of one condition for each participant. All 49 young and 55 older adults were included in the analysis since the data set contained no outliers as confirmed by an outlier analysis using the Tukey method^72^. We analyzed the credibility ratings with a 2 × 3 mixed design (between factor age; within-factor condition with three levels: neutral, positive, negative) permutation *F*-test and followed up on the significant interaction by comparing the average ratings in each condition between young and older adults using three two-sample permutation *t*-tests with adjusted alpha-levels controlling for multiple comparisons using Bonferroni correction (two-sided; α = 0.05/3 = 0.0167).

#### Partial correlations of explicit and implicit cue valence with feedback valence ratings

To assess the link between cue valence and feedback video valence independent of age, we performed partial correlations between cue valence and feedback video valence in each condition using spearman correlation coefficients in jamovi (Version 1.6.23, The jamovi project, 2020). First, we correlated explicit cue valence and feedback video valence and adjusted the alpha-levels to control for multiple comparisons using Bonferroni correction (two-sided; adjusted α = 0.05/3 = 0.0167). Second, we correlated implicit cue valence, measured by the D-scores in the SC-IAT, and feedback video valence and again used Bonferroni correction to control for multiple comparisons (two-sided; adjusted α = 0.05/3 = 0.0167). Since some subjects did not provide all necessary ratings, only 48 young and 51 older adults were included in both analyses.

### Reporting summary

Further information on research design is available in the Nature Research Reporting Summary linked to this article.

### Supplementary information

Reporting summary

## Data Availability

The data that support the findings of this study are openly available at the Center for Open Science (osf.io; 10.17605/OSF.IO/VZ5TM).

## Code availability

Analysis codes are available from the corresponding author upon request, including a formal project outline.
