# Supplementary material for: Effects of positive and negative social feedback on motivation, evaluative learning, and socio-emotional processing
Source: NPJ Sci Learn. 2023 Aug 16;8:28. doi: 10.1038/s41539-023-00178-7 (PMC10432544; doi:10.1038/s41539-023-00178-7)
Supplement: Supplementary file 1 — Reporting summary [file 41539_2023_178_MOESM1_ESM.pdf]

nature portfolio

Corresponding author(s): Alexandra Sobczak
Nico Bunzeck

Last updated by author(s): Oct 7, 2022

# Reporting Summary

Nature Portfolio wishes to improve the reproducibility of the work that we publish. This form provides structure for consistency and transparency in reporting. For further information on Nature Portfolio policies, see our Editorial Policies and the Editorial Policy Checklist.

## Statistics

For all statistical analyses, confirm that the following items are present in the figure legend, table legend, main text, or Methods section.

| n/a | Confirmed | |
|---|---|---|
| ☐ | ☒ | The exact sample size (*n*) for each experimental group/condition, given as a discrete number and unit of measurement |
| ☐ | ☒ | A statement on whether measurements were taken from distinct samples or whether the same sample was measured repeatedly |
| ☐ | ☒ | The statistical test(s) used AND whether they are one- or two-sided<br>*Only common tests should be described solely by name; describe more complex techniques in the Methods section.* |
| ☐ | ☒ | A description of all covariates tested |
| ☐ | ☒ | A description of any assumptions or corrections, such as tests of normality and adjustment for multiple comparisons |
| ☐ | ☒ | A full description of the statistical parameters including central tendency (e.g. means) or other basic estimates (e.g. regression coefficient) AND variation (e.g. standard deviation) or associated estimates of uncertainty (e.g. confidence intervals) |
| ☐ | ☒ | For null hypothesis testing, the test statistic (e.g. $F$, $t$, $r$) with confidence intervals, effect sizes, degrees of freedom and $P$ value noted<br>*Give P values as exact values whenever suitable.* |
| ☐ | ☒ | For Bayesian analysis, information on the choice of priors and Markov chain Monte Carlo settings |
| ☒ | ☐ | For hierarchical and complex designs, identification of the appropriate level for tests and full reporting of outcomes |
| ☐ | ☒ | Estimates of effect sizes (e.g. Cohen's *d*, Pearson's *r*), indicating how they were calculated |

*Our web collection on statistics for biologists contains articles on many of the points above.*

## Software and code

Policy information about availability of computer code

| | |
|---|---|
| Data collection | The SID-Photo study was performed using Cogent Graphics developed by John Romaya at the Laboratory of Neurobiology at the Wellcome Department of Imaging Neuroscience. The SID-Video study was programmed in MATLAB, using the Psychophysics Toolbox extensions (Version 3; Brainard, 1997; Pelli, 1997) since video playback was not possible using Cogent. |
| Data analysis | Statistical analyses were performed with jamovi (Version 1.6.23) and MATLAB 2018b using built-in functions, downloaded content from File Exchange, and custom scripts. |

For manuscripts utilizing custom algorithms or software that are central to the research but not yet described in published literature, software must be made available to editors and reviewers. We strongly encourage code deposition in a community repository (e.g. GitHub). See the Nature Portfolio guidelines for submitting code & software for further information.

## Data

Policy information about availability of data

All manuscripts must include a data availability statement. This statement should provide the following information, where applicable:

- Accession codes, unique identifiers, or web links for publicly available datasets
- A description of any restrictions on data availability
- For clinical datasets or third party data, please ensure that the statement adheres to our policy

The data that support the findings of this study are openly available at the Center for Open Science (osf.io; http://doi.org/10.17605/OSF.IO/VZ5TM).

## Human research participants

Policy information about studies involving human research participants and Sex and Gender in Research.

| | |
|---|---|
| Reporting on sex and gender | We report the gender (shaped by cultural and social circumstances) of our participants as indicated by self-report in a questionnaire including the options “male”, “female”, and “diverse”. Although gender was no variable of interest in our study, we aimed to include 50 % male and 50 % female participants in our sample. However, we were only able to recruit about 30 % male volunteers. |
| Population characteristics | We only included healthy participants, i.e. participants without former or current diseases of the brain and mind (incl. anxiety disorders, depression, schizophrenia, etc.), other serious health problems or current severe mental or physical distress. The sample consists of n = 101 young (mean age 23 years; female = 65) and n = 107 older participants (mean age 64 years; female = 60). |
| Recruitment | We recruited our participants via postings in student mailing lists, newspapers, public spaces, and the database of the institute. The young group mainly includes students from the University of Lübeck and the Technical University Lübeck. Especially in the older group, volunteering for studies could be related to above average openness for new experiences. Furthermore, high performing individuals may be more confident and thus more willing to participate in studies. |
| Ethics oversight | This study was approved by the local ethics committee of the University of Lübeck, Germany. |

Note that full information on the approval of the study protocol must also be provided in the manuscript.

# Field-specific reporting

Please select the one below that is the best fit for your research. If you are not sure, read the appropriate sections before making your selection.

☐ Life sciences ☒ Behavioural & social sciences ☐ Ecological, evolutionary & environmental sciences

For a reference copy of the document with all sections, see nature.com/documents/nr-reporting-summary-flat.pdf

# Behavioural & social sciences study design

All studies must disclose on these points even when the disclosure is negative.

| | |
|---|---|
| Study description | The current study employs an experimental quantitative approach including self-reports and response time data in a mixed design. The between-subject factor was age (two cohorts: young (18-25 years old) and older (50-90 years old) adults) and the within-subject factor was the experimental condition (neutral, positive, and negative social feedback). |
| Research sample | The study includes two age cohorts: young (n = 101; 18-25 years old; mean age 23 years; female = 65) and older (n = 107; 50-90 years old; mean age 64 years; female = 60) adults. The young group mainly includes students from the University of Lübeck and the Technical University Lübeck. Many of our participants are psychology students since they must collect test person hours throughout their studies. The older group mainly consists of volunteers from the city of Lübeck and its surroundings. |
| Sampling strategy | We recruited our participants via postings in student mailing lists, newspapers, public spaces, and the database of the institute. The young group mainly includes students from the University of Lübeck and the Technical University Lübeck while the older group mainly consists of volunteers from the city of Lübeck and its surroundings. The sample size was determined by previous work from the lab and other research groups with similar designs. |
| Data collection | We used a custom questionnaire to assess the inclusion criteria and further demographic variables. The Montreal Cognitive Assessment (Nasreddine et al., 2005) was used in the original pen and paper version. The experimental tasks were performed on a computer. Audio of the video feedback was presented via over-ear headphones. The participant performed the experimental tasks in a separate test room while the experimenter supervised the experiment from a control room. The experimenter was not blind to the study hypothesis but followed a standardized instruction protocol to avoid potential bias. Since all experimental conditions were tested in each subject (within-subject factor), the experimenter was not blind to the experimental condition. |
| Timing | Data collection for the young sample of the SID-Photo study started in March 2016 and stopped in May 2016. Data for the older sample of the SID-Photo study was collected from February 2019 until April 2019. Data for the SID-Video study (young and older sample) was collected from October 2020 until June 2021. |
| Data exclusions | One young and three older participants of the SID-Video study were excluded due to fulfilling pre-established exclusion criteria (the young participant reported depression in clinical history, one older participant reported a stroke in clinical history and two older participants scored below 22 in the MoCA). Final sample sizes for each task can vary due to technical problems in data acquisition and are therefore be reported separately for each task in the manuscript. For each analysis, outliers were identified using the Tukey method (Tukey, 1977) and removed as reported in the manuscript. |

| | |
|---|---|
| Non-participation | Due to technical problems in data acquisition, some participants did not perform all experimental tasks. The final sample sizes for each task are reported in the manuscript. |
| Randomization | The study used a mixed design with the factors age and condition. The experimental conditions were varied within subjects and were therefore not randomly allocated. |

# Reporting for specific materials, systems and methods

We require information from authors about some types of materials, experimental systems and methods used in many studies. Here, indicate whether each material, system or method listed is relevant to your study. If you are not sure if a list item applies to your research, read the appropriate section before selecting a response.

## Materials & experimental systems

| n/a | Involved in the study |
|---|---|
| ☒ | ☐ Antibodies |
| ☒ | ☐ Eukaryotic cell lines |
| ☒ | ☐ Palaeontology and archaeology |
| ☒ | ☐ Animals and other organisms |
| ☒ | ☐ Clinical data |
| ☒ | ☐ Dual use research of concern |

## Methods

| n/a | Involved in the study |
|---|---|
| ☒ | ☐ ChIP-seq |
| ☒ | ☐ Flow cytometry |
| ☒ | ☐ MRI-based neuroimaging |
